# Persistent spatial hotspots and socioeconomic inequalities of snakebite envenoming in Peru: A nationwide spatiotemporal analysis, 2010–2025

**DOI:** 10.1371/journal.pntd.0014747

**Published:** 2026-09-24

**Authors:** Malena Grados Vásquez, Juan Carlos Guerra Blas, Anthony Salcedo-Prudencio, Thayane Santos Siqueira, Wuelton Monteiro, John Alexanders Amaya Parra, Flor Ernestina Martinez-Espinosa

**Affiliations:** 1 Department of Biochemistry and Immunology, Institute of Biological Sciences, Federal University of Minas Gerais (UFMG), Belo Horizonte, Minas Gerais, Brazil; 2 Division of Impacts, Adaptation and Vulnerabilities (DIIAV), National Institute for Space Research (INPE), São José dos Campos, São Paulo, Brazil; 3 Health Sciences Graduate Program, Federal University of Sergipe, Aracaju, Sergipe, Brazil; 4 Universidade do Estado do Amazonas (UEA), Manaus, Amazonas, Brazil; 5 Fundação de Medicina Tropical Dr. Heitor Vieira Dourado (FMT-HVD), Manaus, Amazonas, Brazil; 6 Duke Global Health Institute, Duke University, Durham, North Carolina, United States of America; 7 Instituto Leônidas e Maria Deane (ILMD/Fiocruz Amazônia), Manaus, Amazonas, Brazil; Universidad Regional Amazónica IKIAM: Universidad Regional Amazonica IKIAM, ECUADOR

## Abstract

**Background:**

Snakebite envenoming remains a major neglected tropical disease worldwide, disproportionately affecting rural and socioeconomically vulnerable populations in tropical and subtropical regions. In Peru, snakebite burden is strongly concentrated in Amazonian territories, yet nationwide evidence on its long-term spatial dynamics and socioeconomic determinants remains limited.

**Methodology:**

This study provides the first multiscale spatiotemporal assessment of snakebite envenoming incidence in Peru between 2010 and 2025. National surveillance data were analyzed at departmental and district levels to evaluate temporal trends, spatial clustering, hotspot persistence, ecological suitability, and socioeconomic associations. Spatial autocorrelation was assessed using Global Moran’s I and Local Indicators of Spatial Association (LISA), while associations between Human Development Index (HDI) and snakebite envenoming incidence were evaluated across Peruvian macroregions.

**Findings:**

A total of 32,942 snakebite envenoming cases were reported during the study period, with more than 60% concentrated in Amazonian departments, particularly Loreto, San Martín, and Ucayali. Snakebite incidence exhibited significant spatial autocorrelation throughout the study period (Moran’s I = 0.53–0.61; *p* < 0.05), revealing a non-random and spatially structured distribution of risk. LISA analyses identified persistent hotspot districts concentrated mainly in the northern and central Peruvian Amazon, forming a stable corridor of elevated incidence over time. Persistent hotspots spatially overlapped with areas of high environmental suitability for medically important venomous snakes, particularly *Bothrops atrox*, and elevated potential species richness. Socioeconomic analyses revealed marked regional heterogeneity, with a significant moderate negative correlation between HDI and snakebite incidence in the Northern Amazon (Spearman’s ρ = −0.42; *p* < 0.001), indicating higher burden in socioeconomically vulnerable districts.

**Conclusions:**

These findings demonstrate that snakebite envenoming in Peru is spatially persistent, ecologically structured, and closely associated with socioeconomic inequalities in Amazonian regions. Identifying persistent high-risk areas may support geographically targeted surveillance, optimized antivenom allocation, and territorially adapted public health interventions.

## Author summary

Snakebite envenoming is a major public health problem that mainly affects poor rural populations living in tropical regions. In Peru, most snakebite cases occur in the Amazon, where many communities depend on agriculture, fishing, forestry, and river transport, often with limited access to healthcare and antivenom treatment. Despite this, little was known about how snakebite risk has changed across Peru over time or whether the same high-risk areas persist year after year. In this study, we analyzed national snakebite surveillance data from 2010 to 2025 to identify long-term spatial patterns of snakebite envenoming in Peru. We found that snakebite risk is not randomly distributed but concentrated in persistent hotspot districts located mainly in the northern and central Amazon. These hotspots also overlapped with areas environmentally suitable for medically important venomous snakes and with districts presenting lower levels of human development. Our findings show that snakebite envenoming in Peru is strongly linked to both environmental conditions and social inequalities. Identifying persistent high-risk areas may help improve epidemiological surveillance, prevention campaigns, and antivenom distribution in vulnerable Amazonian populations.

## Introduction

Snakebite envenoming (SBE) is a neglected tropical disease that disproportionately affects rural and impoverished populations in tropical and subtropical regions worldwide [1]. Globally, an estimated 1.8–2.7 million cases of envenoming and 81,000–138,000 deaths occur annually, along with more than 400,000 cases resulting in permanent physical disability and long-term socioeconomic consequences [1,2]. Despite the availability of effective antivenom therapy, limited healthcare infrastructure, delayed access to treatment, insufficient antivenom distribution, and structural social inequalities continue to hinder adequate management, particularly in low-resource rural settings [3]. Consequently, SBE is increasingly recognized not only as a medical emergency, but also as a disease strongly associated with poverty, territorial vulnerability, and health inequities [2,4,5].

In tropical forest regions, SBE burden is closely associated with livelihoods that involve direct interaction with natural environments, including agriculture, forestry, fishing, hunting, and extractive activities [6–9]. Indigenous peoples, small-scale farmers, riverine communities, and forest-dependent populations are among the groups at highest risk due to increased occupational and environmental exposure to venomous snakes [10–12]. In the Amazon basin, favorable ecological conditions for medically important snake species, combined with ongoing environmental transformation, deforestation, agricultural expansion, and intensified human occupation contribute to geographically heterogeneous and persistent SBE risk [13–15].

In the Americas, more than 57,000 SBEs are estimated to occur annually [16]. In Peru, national surveillance systems report approximately 1,700–2,800 SBE cases annually, corresponding to an incidence of approximately 5–8 cases per 100,000 inhabitants based on records from the National Center for Epidemiology, Prevention and Disease Control of the Peruvian Ministry of Health [17]. However, the true burden is likely underestimated, particularly in remote Amazonian territories where healthcare access remains limited and traditional medicine frequently represents the first therapeutic response [12,18].

The epidemiology of SBE in Peru is strongly influenced by the country’s marked ecological and biogeographic heterogeneity, which encompasses the Pacific coastal desert, the Andean mountain range, and the Amazon rainforest. Peru harbors a high diversity of venomous snake species, primarily belonging to the families Viperidae and Elapidae [19]. Among them, *Bothrops atrox* (“jergón” or fer-de-lance) is the principal medically important species and is responsible for most reported envenomings in the Peruvian Amazon [20,21]. Other medically important species, including *Bothrops bilineatus*, *Lachesis muta*, *Bothrops pictus*, and *Bothrops barnetti*, also contribute to regional patterns of SBE occurrence across Amazonian and coastal areas of the country [22–24]. SBE distribution is influenced by climatic, environmental, ecological, and socioeconomic drivers, including rainfall variability, temperature, deforestation, agricultural expansion, and land-use change [4,13,25,26]. Furthermore, climate change and global environmental transformations may alter the future geographic distribution of venomous snakes and expand SBE risk hotspots in vulnerable tropical regions [27,28].

Recent advances in spatial epidemiology and geographic information systems (GIS) have enabled the identification of spatial clustering, hotspot persistence, and geographic heterogeneity of SBE burden across tropical regions [29–31]. Spatial analytical approaches such as Moran’s I and Local Indicators of Spatial Association (LISA) have been increasingly applied to identify statistically significant transmission clusters and persistent high-risk areas in tropical regions [31–33]. Nevertheless, most previous studies conducted in Peru have been restricted to hospital-based case series, local epidemiological descriptions, or short temporal windows, providing limited understanding of long-term spatial dynamics and territorial persistence of SBE [34–36]. Despite the well-recognized concentration of cases in Amazonian departments such as Loreto, Ucayali, San Martín, and Huánuco, nationwide evidence integrating spatiotemporal epidemiology, hotspot persistence, ecological suitability, and socioeconomic vulnerability remains scarce.

Understanding whether SBE risk in Peru is spatially persistent rather than randomly distributed has important implications for public health planning, epidemiological surveillance, antivenom allocation, and territorially targeted prevention strategies. In geographically isolated Amazonian districts, where environmental suitability for venomous snakes overlaps with socioeconomic vulnerability and reduced healthcare accessibility, identifying persistent hotspots may contribute to improving resource prioritization and strengthening healthcare responses in historically underserved territories.

Based on the marked ecological and socioeconomic heterogeneity across Peru, we hypothesized that SBE incidence would be spatially structured rather than randomly distributed, and that persistent hotspots would be associated with high environmental suitability for medically important venomous snakes, lower levels of human development, and greater socioeconomic vulnerability.

Therefore, this study provides the first nationwide multiscale spatiotemporal assessment of SBE in Peru between 2010 and 2025. Specifically, we aimed to: (i) characterize temporal trends and geographic patterns of SBE incidence at departmental and district levels; (ii) identify statistically significant spatial clusters and persistent hotspots of SBE incidence using spatial autocorrelation analyses; (iii) evaluate the spatial overlap between persistent SBE hotspots and the environmental suitability and richness of medically important venomous snakes; and (iv) assess the relationship between socioeconomic inequalities and SBE incidence across Peruvian macroregions.

## Materials and methods

### Study area

Peru covers approximately 1.28 million km² and is administratively divided into 24 departments, one Constitutional Province (Callao), and 1,891 districts, encompassing highly heterogeneous geographic and ecological regions, including the Pacific coastal desert, the Andean mountain range, and the Amazon rainforest [37] (Fig 1). Natural regions were classified according to the framework proposed by Pulgar Vidal [38], including Costa, Sierra, Ceja de Selva, Selva Alta, and Selva Baja. Marked environmental gradients driven by altitude, topography, and climate strongly influence biodiversity, land use, human occupation patterns, and the geographic suitability of venomous snakes across the country.

**Fig 1:**
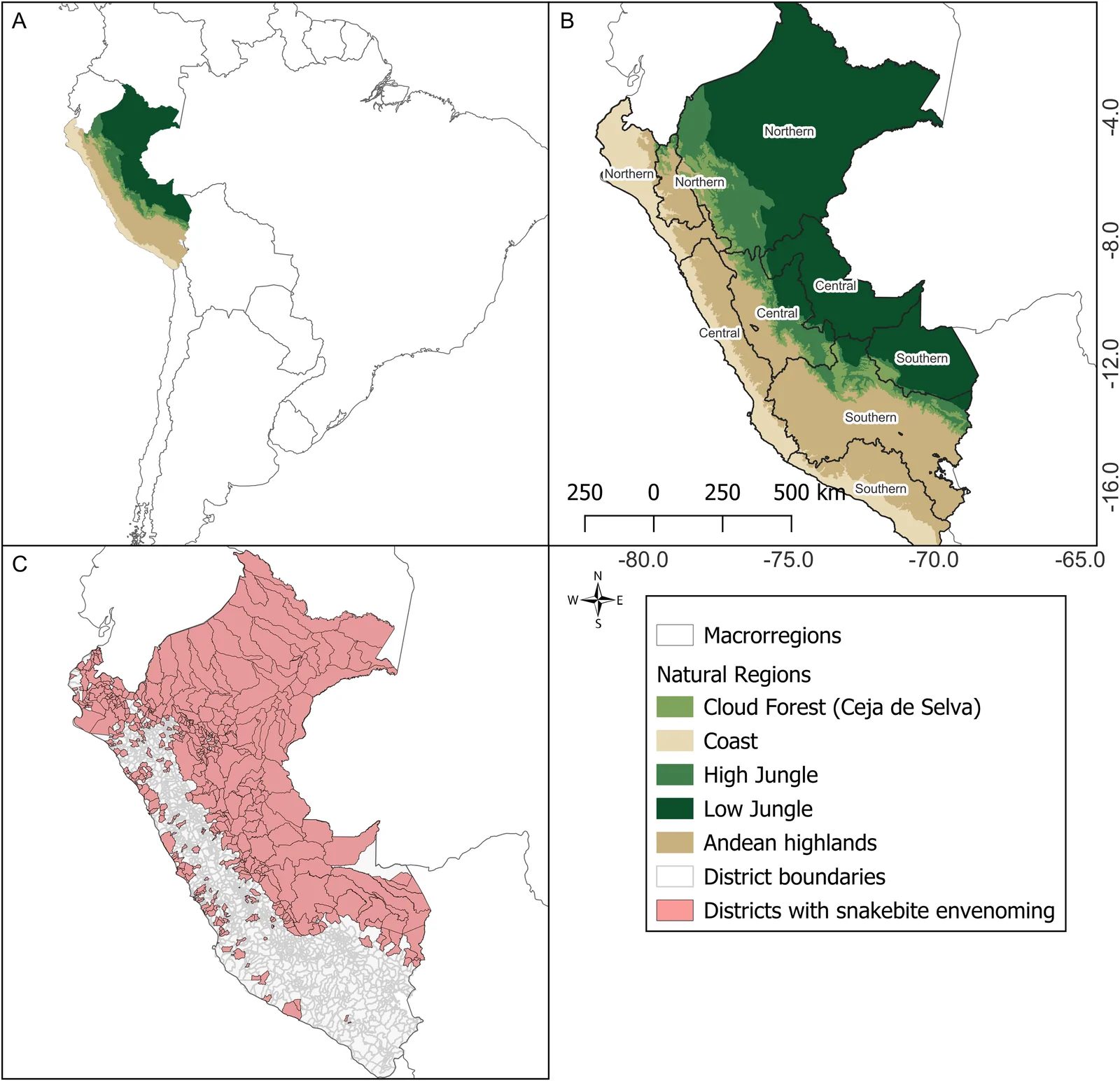
Study area and spatial units used in the analysis of snakebite envenoming in Peru, 2010–2025. (**A)** Geographic location of Peru within South America. **(B)** Natural regions and macroregions of administrative departments of Peru. Natural regions were adapted from the classification proposed by Pulgar Vidal [38], including Costa, Sierra, Ceja de Selva, Selva Alta, and Selva Baja. Department codes correspond to the official administrative divisions of Peru. **(C)** District-level spatial units included in the study. Districts reporting at least one SBE case during the study period are highlighted in pink, whereas districts without reported cases are shown in light gray. Administrative boundaries were obtained from the official district-level shapefiles produced by the Instituto Geográfico Nacional del Perú (IGN) and distributed through the Peruvian National Open Data Platform [39]. The administrative boundary dataset is publicly available under the Open Data Commons Attribution License (ODC-By). Country boundaries for South America were obtained from Natural Earth (Admin 0–Countries, 1:110m) [40]. Natural Earth data are in the public domain.

The coastal region (Costa) is predominantly arid to semi-arid, whereas the Andean region (Sierra) is characterized by strong altitudinal variability and marked climatic seasonality. In contrast, the Amazonian region (Selva), including both Selva Alta and Selva Baja, presents humid tropical conditions, high annual rainfall, elevated temperatures, and extensive forest cover [41–43]. These environmental conditions, combined with agricultural expansion, extractive activities, and the presence of riverine and forest-dependent populations, are associated with increased opportunities for human–snake encounters, particularly within the Amazon basin.

For temporal and socioeconomic analyses, departments were grouped into analytical macroregions based on geographic proximity, ecological characteristics, and regional epidemiological patterns. These analytical macroregions do not represent official administrative divisions but were established to facilitate comparisons among areas with broadly similar environmental and epidemiological contexts. Three major geographic domains (Amazon, Andes, and Coast) were subdivided into northern, central, and southern sectors. The complete classification of departments included within each macroregion is provided in S1 Table.

### Study design and overview

An ecological multiscale study was conducted to analyze the spatial and temporal patterns of snakebite envenoming (SBE) in Peru between 2010 and 2025. Epidemiological data were aggregated at both departmental and district levels, allowing the assessment of geographic heterogeneity across multiple spatial scales. Throughout this manuscript, snakebite envenoming is hereafter referred to as SBE.

The study comprised four complementary analytical components: (i) descriptive temporal and spatial analyses of SBE incidence at national, departmental, and district levels; (ii) assessment of spatial autocorrelation and identification of statistically significant spatial clusters (hotspots and coldspots) using Global Moran’s I and Local Indicators of Spatial Association (LISA); (iii) evaluation of the temporal persistence of High–High hotspot districts across predefined study periods; and (iv) integration of ecological suitability models and socioeconomic indicators to explore the environmental and social context associated with persistent SBE hotspots.

The methodological workflow included epidemiological data acquisition and harmonization, incidence estimation, temporal trend analysis, seasonal analysis of monthly incidence patterns, spatial autocorrelation analysis, ecological suitability assessment using species distribution models (SDMs) for medically important venomous snakes, estimation of potential venomous snake richness, evaluation of ecological overlap between predicted species distributions and persistent SBE hotspots, and socioeconomic analyses based on district-level Human Development Index (HDI) indicators.

### Epidemiological data

SBE case data were obtained from the Peruvian Ministry of Health (MINSA) through an official information request submitted to the National Center for Epidemiology, Prevention and Disease Control (CDC–MINSA) under the Peruvian Transparency and Access to Public Information Law (Ley N° 27806). The dataset was provided by the Directorate of Public Health Surveillance (Dirección de Vigilancia en Salud Pública), the technical unit responsible for the management and dissemination of national epidemiological surveillance data.

The national surveillance system compiles SBE notifications reported by healthcare facilities throughout the country through the official epidemiological reporting platform. The database consisted of annual aggregated SBE case counts at both departmental and district levels covering the period from 2010 to 2025. Reported cases correspond to individuals who sought medical care and were officially registered within the national health surveillance system. Cases included in the database correspond to snakebite envenoming events notified through the national epidemiological surveillance system according to the reporting criteria established by the Peruvian Ministry of Health [17].

As with other passive surveillance systems, the reported number of cases may underestimate the true burden of SBE because individuals who do not seek medical care or are not captured by the surveillance network are not included in the database [2].

Because the analyses were based exclusively on secondary aggregated public health surveillance data without individual patient identification, ethical committee approval and informed consent were not required according to Peruvian regulations governing the use of anonymized epidemiological data. All data were handled in accordance with applicable data protection and confidentiality regulations, and no personally identifiable information was accessed at any stage of the study.

### Population data and incidence estimation

SBE incidence was calculated as the annualized number of reported cases per 100,000 inhabitants using the total resident population as denominator. Temporal analyses were standardized into three study periods (2010–2015, 2016–2020, and 2021–2025) to facilitate spatiotemporal comparisons. These multi-year periods were defined for analytical purposes to reduce the influence of interannual variability, improve the stability of district-level incidence estimates, and facilitate the identification and comparison of persistent spatial clustering patterns. The selected temporal groupings do not correspond to specific epidemiological or public health intervention milestones. Records with incomplete temporal or spatial information were excluded from period-based analyses. For each study period, total cases were divided by the number of years included in the respective interval and standardized using the corresponding population anchor year: 2015 for 2010–2015, 2020 for 2016–2020, and 2025 for 2021–2025. Incidence was therefore estimated as:


$$\begin{array}{c} \text{Incidence}=\left(\frac{\text{cases in period}/\text{number of years}}{\text{population}}\right)\times 100000 \end{array}$$

This annualization procedure enabled direct comparison among study periods of unequal duration while minimizing bias associated with differences in observation time. Population denominators were derived from official demographic projections provided by the National Institute of Statistics and Informatics of Peru (INEI) [37]. The resulting incidence estimates provided a standardized population-based measure suitable for comparisons across districts and study periods for epidemiological surveillance and public health planning purposes. Nevertheless, exposure to SBE is inherently heterogeneous and influenced by occupational, environmental, and behavioral factors, particularly among rural populations engaged in agriculture, forestry, fishing, and extractive activities. Consequently, the estimated incidence values reflect population-level risk rather than incidence restricted to specific high-exposure groups.

### Socioeconomic data

District-level Human Development Index (HDI) data were obtained from the United Nations Development Programme (UNDP) Peru through the official database “Anexo 1: IDH ajustado por desigualdad 2017–2024 a nivel distrital” [44]. The HDI is a composite indicator that measures average achievement in three key dimensions of human development: a long and healthy life, access to knowledge, and a decent standard of living. The database includes annual district-level estimates of the inequality-adjusted Human Development Index (IHDI) for all Peruvian districts between 2017 and 2024.

The socioeconomic analysis was restricted to the 2017–2024 period because district-level HDI estimates with standardized nationwide coverage were only consistently available for these years in the official UNDP Peru database. Consequently, associations between socioeconomic conditions and SBE incidence were evaluated using the temporally overlapping period between HDI availability and epidemiological records.

To evaluate the relationship between socioeconomic conditions and SBE burden, mean HDI values were calculated for each district considering the entire 2017–2024 period. District-level HDI data were subsequently integrated with mean annual SBE incidence estimates using official UBIGEO geographic codes.

### Species distribution models and ecological suitability

Environmental suitability for medically important venomous snake species in Peru was assessed using species distribution models (SDMs), a widely applied framework for estimating the potential geographic distribution of species based on environmental suitability and ecological niche relationships [45,46]. We used previously developed species distribution models available through VenomMaps [47]. Continuous and thresholded SDM raster outputs were downloaded from the VenomMaps web application (https://rhettrautsaw.github.io/VenomMaps/) and subsequently processed at the district level. The VenomMaps dataset and SDM outputs are available under the Creative Commons Attribution 4.0 International License (CC BY 4.0).

The analysis included medically important venomous snake species known to occur in Peru, including *Bothrops atrox*, *Bothrops bilineatus*, *Bothrops barnetti*, *Bothrops pictus*, *Bothrops oligolepis*, *Bothrocophias microphthalmus*, and *Lachesis muta*. Continuous environmental suitability rasters and binary suitability layers were obtained at approximately 1-km spatial resolution from the VenomMaps database. Raster layers were spatially aggregated to district-level administrative units using the R package *terra* [48].

For each district and species, summary metrics of environmental suitability were calculated, including mean, median, maximum, standard deviation, and coefficient of variation values. Median suitability values were selected for the main analyses because they provide a robust measure of central tendency that is less sensitive to extreme suitability values and isolated outliers.

The species distribution models used in this study represent long-term potential environmental suitability rather than annual species distributions. Consequently, SDM outputs were not intended to reproduce temporal changes in snake occurrence during 2010–2025, but rather to provide a spatially explicit representation of areas environmentally suitable for medically important venomous snakes. These suitability maps were therefore used as static ecological layers to evaluate spatial overlap with persistent SBE hotspots identified across the study period.

### Temporal analysis

At the national level, temporal changes in annual SBE incidence were evaluated using continuous segmented linear regression. Candidate models containing zero, one, or two breakpoints were fitted. Starting values supplied to the segmented models were used only to facilitate model convergence and were not treated as fixed change points. Evidence of a change in slope was initially evaluated using Davies’ test. Candidate models were compared using the corrected Akaike Information Criterion (AICc) and Bayesian Information Criterion (BIC), and the model with the lowest AICc was selected. Estimated breakpoints, segment-specific slopes, standard errors, and 95% confidence intervals were obtained from the selected model.

Both at national and macroregional levels, monotonic temporal trends were assessed using Annual Percent Change (APC) estimates and the non-parametric Mann–Kendall trend test [49,50]. APC values were calculated from annual incidence series to estimate the magnitude and direction of temporal changes over time, whereas Mann–Kendall statistics were used to evaluate the significance of monotonic trends while minimizing sensitivity to non-normality and interannual variability. Analyses were conducted separately for Amazonian, Andean, and Coastal macroregions.

Seasonal patterns were evaluated by calculating mean monthly SBE incidence and corresponding standard deviations across the 2010–2025 period. Monthly incidence profiles were used to characterize recurrent intra-annual variability and identify seasonal peaks in SBE incidence.

### Spatial analyses

All spatial datasets were harmonized to a common spatial reference system and aggregated according to district boundaries. Spatial analyses were conducted at the district level to quantify spatial dependence in SBE incidence and identify statistically significant clustering patterns, following widely applied approaches in spatial epidemiology [29,51].

To ensure compatibility between epidemiological records and cartographic databases, administrative names and geographic identifiers were standardized prior to spatial analyses. Data-cleaning procedures included correction of encoding inconsistencies, harmonization of accents and special characters, removal of duplicated spaces, and correction of known spelling inconsistencies between surveillance records and shapefiles. District-level linkage between epidemiological and spatial datasets was performed using official UBIGEO geographic codes. Quality-control procedures were subsequently applied to verify matching rates between surveillance records and district polygons and to minimize potential spatial misclassification.

All district-level spatial analyses were conducted using the complete set of 1,891 districts in Peru, including districts reporting zero SBE incidence during a given study period, to preserve the national spatial structure and avoid biases associated with excluding zero-incidence areas.

Temporal analyses were standardized into three predefined study periods (2010–2015, 2016–2020, and 2021–2025), and records with incomplete temporal or spatial information were excluded from period-based analyses. Spatial datasets were reprojected to WGS 84 / UTM Zone 18S (EPSG:32718) to ensure consistency in spatial analyses and enable Euclidean distance calculations in meters.

Spatial relationships among districts were modeled using a symmetrized k-nearest-neighbors (k-NN) spatial weights matrix (k = 4) based on district centroids [52]. All weights were row-standardized prior to analysis. Global spatial autocorrelation was assessed using Moran’s I [53], whereas local spatial dependence was evaluated using Local Indicators of Spatial Association (LISA) based on Local Moran’s I [54]. Statistical significance was assessed using 999 Monte Carlo permutations, adopting *p* < 0.05 as the primary significance threshold. LISA analyses enabled the identification of statistically significant High–High (HH) clusters (hotspots), Low–Low (LL) clusters (coldspots), and spatial outliers (High–Low and Low–High districts).

To evaluate the robustness of hotspot identification, sensitivity analyses were conducted using alternative spatial weights matrices, including k-NN specifications with k = 6 and k = 8, and a first-order Queen contiguity matrix. Global Moran’s I values and LISA cluster classifications obtained under alternative weighting schemes were compared with those derived from the primary k = 4 model. LISA analyses were performed independently for each of the three study periods (2010–2015, 2016–2020, and 2021–2025) using period-specific annualized district-level incidence. Each district therefore received a separate LISA classification for each study period.

After completing the three independent period-specific LISA analyses, hotspot persistence was evaluated by counting the number of study periods in which each district was classified as a statistically significant High–High (HH) cluster. Districts were categorized as HH in 0, 1, 2, or 3 periods. Persistent hotspots were defined as districts classified as HH in at least two periods, whereas transient hotspots were classified as HH in only one period.

To complement the hotspot persistence analysis, long-term district-level SBE incidence was calculated as the mean of the annualized incidence estimates across the three study periods (2010–2015, 2016–2020, and 2021–2025). Percentile thresholds were derived from the national distribution of long-term mean incidence across all 1,891 districts, including districts with zero incidence. Districts were classified into four mutually exclusive categories: < P75, ≥P75 to <P90, ≥P90 to <P95, and ≥P95. A cross-tabulation between hotspot persistence and incidence percentile categories was then performed to examine the relationship between long-term disease burden and hotspot persistence.

All data processing, statistical analyses, and spatial analyses were conducted in R version 4.2.3 using packages including *sf*, *spdep*, *dplyr*, *tidyr*, *ggplot2*, and *patchwork*.

### Potential species richness and integration with persistent hotspots

Binary suitability layers were used to estimate potential species presence within each district. District-level potential richness was calculated as the sum of medically important venomous snake species presenting at least one environmentally suitable raster cell within district boundaries, generating a composite richness layer representing the potential overlap of venomous snake species across Peru. Potential richness represents environmentally suitable areas predicted by species distribution models and should not be interpreted as confirmed species occurrence [45]. The species distribution models were not developed, fitted, or recalibrated in the present study. We used previously developed and validated predictions obtained from the VenomMaps repository. Consequently, model performance metrics and variable-importance estimates were not generated in this study; details of model development, predictors, and validation are available in the original publication by Rautsaw et al. (2022) [47].

To evaluate the ecological context of SBE persistence, district-level richness and environmental suitability metrics were spatially integrated with persistent SBE hotspots identified through Local Indicators of Spatial Association (LISA). Persistent hotspots were defined as districts classified as High–High clusters in at least two study periods (2010–2015, 2016–2020, and 2021–2025).

Differences in richness between persistent High–High (HH) LISA hotspots and other districts, as well as between districts within the upper incidence percentiles (P75, P90, and P95) and the remaining districts, were assessed using Wilcoxon rank-sum tests. Spatial analyses, ecological metrics, statistical analyses, and map generation were performed in R version 4.2.3 using the packages *terra*, *sf*, *dplyr*, *ggplot2*, and *patchwork*.

### Socioeconomic analysis

Associations between socioeconomic development and SBE incidence were evaluated at the district level across Peruvian macroregions. Because district-level SBE incidence exhibited marked asymmetry, zero inflation, and extreme values, particularly in sparsely populated districts, districts with a mean population below 1,000 inhabitants were excluded to reduce statistical instability. Districts with missing HDI or SBE incidence data were also excluded. Mean district-level HDI and annualized SBE incidence were calculated for the 2017–2024 period. The log1p transformation of incidence was used for graphical visualization, whereas inferential analyses were conducted using the original incidence values. Because the incidence distribution deviated markedly from normality, associations were evaluated separately for each macroregion using two-sided Spearman rank correlations, which do not require normally distributed data and assess monotonic relationships. For each macroregion, Spearman’s correlation coefficient (ρ), 95% confidence interval, p-value, and number of districts were reported, with confidence intervals estimated using 5,000 non-parametric bootstrap resamples. Relationships between HDI and SBE incidence were visualized using scatterplots with locally estimated scatterplot smoothing (LOESS) curves [55] to explore potential non-linear patterns without imposing a predefined functional form. Estimates from macroregions with small sample sizes or limited incidence variability were interpreted cautiously. All analyses and visualizations were performed in R version 4.2.3 using the packages *dplyr*, *ggplot2*, and *stats*.

## Results

A total of 32,942 snakebite envenoming (SBE) cases were included in this nationwide analysis, based on all officially reported cases recorded by the Peruvian national surveillance system between 2010 and 2025. This corresponds to a mean annual number of 2,059 reported cases (SD = 371). The temporal and spatial analyses presented below are based on this complete nationwide surveillance dataset.

Results are presented following the analytical sequence described in the Methods: temporal patterns, geographic distribution, spatial autocorrelation and hotspot persistence, ecological suitability and potential species richness, and socioeconomic associations.

### Temporal patterns of SBE incidence in Peru (2010–2025)

Following the national and macroregional temporal analyses, SBE incidence in Peru showed an overall declining trend between 2010 and 2025, although with marked interannual variability (Fig 2A). Annual incidence decreased from 7.18 cases per 100,000 inhabitants in 2010 (2,282 reported cases) to 6.15 cases per 100,000 inhabitants in 2025 (2,115 cases). The highest incidence was recorded in 2011 (7.23 per 100,000 inhabitants), whereas the lowest values occurred during 2020 and 2021, with 5.10 and 4.84 cases per 100,000 inhabitants, respectively.

**Fig 2:**
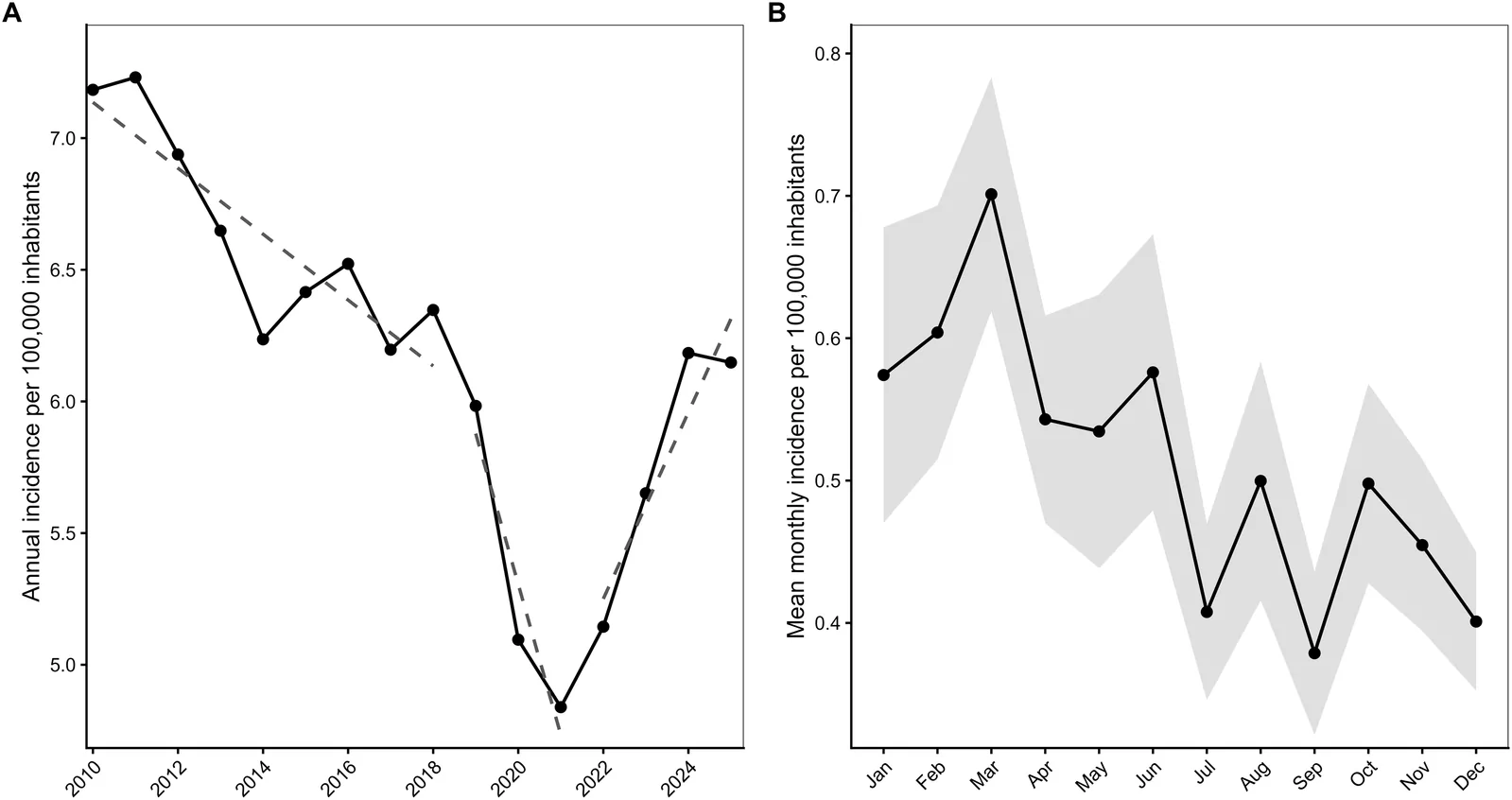
National temporal dynamics of SBE incidence in Peru, 2010–2025. **(A)** Annual SBE incidence per 100,000 inhabitants. The dashed line represents the fitted continuous segmented regression, and the dotted vertical lines indicate the statistically estimated breakpoints at approximately 2018.97 and 2020.48. **(B)** Mean monthly SBE incidence per 100,000 inhabitants; shaded areas represent ±1 standard deviation across years (2010–2025).

Continuous segmented linear regression provided evidence of two changes in the temporal slope of national SBE incidence (Fig 2A). The estimated breakpoints occurred at approximately 2018.97 and 2020.48. Before the first breakpoint, incidence showed a gradual decline of -0.125 cases per 100,000 inhabitants per year (95% CI: -0.182 to -0.068). Between the two breakpoints, the decline became substantially steeper (slope = -0.888; 95% CI: -1.514 to -0.261). After the second breakpoint, the fitted trend changed direction and incidence increased by +0.366 cases per 100,000 inhabitants per year (95% CI: 0.226 to 0.506), approaching pre-2020 levels by 2024–2025. The two-breakpoint model showed greater support than the one-breakpoint and simple linear models according to AICc (14.2, 18.3, and 28.5, respectively). However, because the interval between the two estimated breakpoints contained only two annual observations, the magnitude of the intermediate decline should be interpreted cautiously.

Monthly analyses revealed a consistent seasonal pattern in SBE incidence throughout the study period (Fig 2B). Incidence was consistently higher during the first months of the year, particularly in March, which presented the highest mean monthly incidence (0.70 cases per 100,000 inhabitants), followed by February (0.60) and June (0.57). In contrast, lower incidence values were observed during the austral dry-season months, especially in September (0.38), December (0.40), and July (0.41 cases per 100,000 inhabitants). Seasonal variability was also greater during the rainy-season months, as indicated by higher interannual standard deviations between January and June, whereas lower variability was observed during the dry season.

These temporal patterns were further supported by APC and Mann–Kendall analyses. National SBE incidence showed a significant overall decline between 2010 and 2025 (APC = −1.76% per year; Mann–Kendall τ=−0.65, *p* < 0.001). Similar decreasing trends were observed across Amazonian macroregions, particularly in the Southern Amazon (APC = −4.19% per year, *p* = 0.011) and Northern Amazon (APC = −2.01% per year, *p* < 0.001), indicating a progressive reduction in incidence over time. Nevertheless, despite these declining trends, Amazonian macroregions consistently maintained the highest SBE incidence rates nationwide throughout the study period (S1 Fig and S2 Table).

Temporal dynamics in the Andes and coastal regions were comparatively more heterogeneous. A significant decline was observed in the Northern Andes (APC = −7.14% per year, *p* = 0.002), whereas incidence in the Central and Southern Andes remained relatively stable or showed slight increases over time. In contrast, coastal macroregions consistently exhibited the lowest SBE incidence nationwide and did not present significant monotonic temporal trends during the study period (S1 Fig and S2 Table).

### Spatial distribution of SBE incidence

Based on the department-level incidence estimates calculated for the three predefined study periods, the spatial distribution of SBE burden was markedly heterogeneous across Peru.

The spatial distribution of SBE burden was markedly heterogeneous across the country, with a strong concentration in Amazonian departments (Table 1; Fig 3). Between 2010 and 2025, a total of 32,942 SBE cases were reported in Peru, corresponding to a mean annual number of 2,059 cases (SD = 371). Loreto recorded the highest number of reported cases (9,484; 28.8% of total cases), followed by San Martín (6,561) and Ucayali (4,092). Together, these three departments accounted for more than 60% of all SBE cases reported during the study period. Other Amazon-influenced departments, including Amazonas, Junín, Huánuco, Pasco, and Madre de Dios, also consistently exhibited higher case counts than coastal and southern regions.

**Table 1:** Department-level snakebite envenoming burden and average annual incidence rates across Peru, 2010–2025.

Department	Total Cases	Average annual cases			Incidence (per 100,000)		
		2010–2015	2016–2020	2021–2025	2010–2015	2016–2020	2021–2025
Loreto	9484	662	531	572	63.7	51.7	53.5
San Martín	6561	416	417	396	49.4	46.4	42.1
Ucayali	4092	281	242	239	56.8	41.1	37.0
Amazonas	2866	197	163	173	46.7	38.2	39.6
Junín	2378	128	160	162	9.5	11.8	11.7
Huánuco	1945	138	124	100	16.0	16.3	13.5
Cusco	1153	84	81	49	6.4	6.0	3.5
Pasco	1014	67	55	67	22.1	20.2	25.4
Madre de Dios	892	59	57	51	42.7	32.7	24.2
Piura	709	44	48	44	2.3	2.3	2.0
Ayacucho	356	13	28	28	1.8	4.2	4.2
Cajamarca	341	29	22	12	1.9	1.5	0.8
Lima	295	22	17	16	0.2	0.2	0.1
La Libertad	256	21	17	10	1.1	0.8	0.4
Puno	238	6	15	26	0.4	1.2	2.1
Lambayeque	105	8	6	6	0.6	0.4	0.4
Tumbes	102	8	4	7	3.2	1.7	2.3
Áncash	77	7	3	5	0.6	0.2	0.4
Huancavelica	43	4	3	1	0.8	0.8	0.3
Ica	23	0	1	4	0.0	0.1	0.3
Arequipa	9	1	0	1	0.1	0.0	0.1
Apurímac	2	0	0	0	0.0	0.1	0.0
Callao^*^	1	0	0	0	0.0	0.0	0.0
Total	32942	2192	1994	1968	326.2	277.9	264.1

* Callao corresponds to the Constitutional Province of Callao.

**Fig 3:**
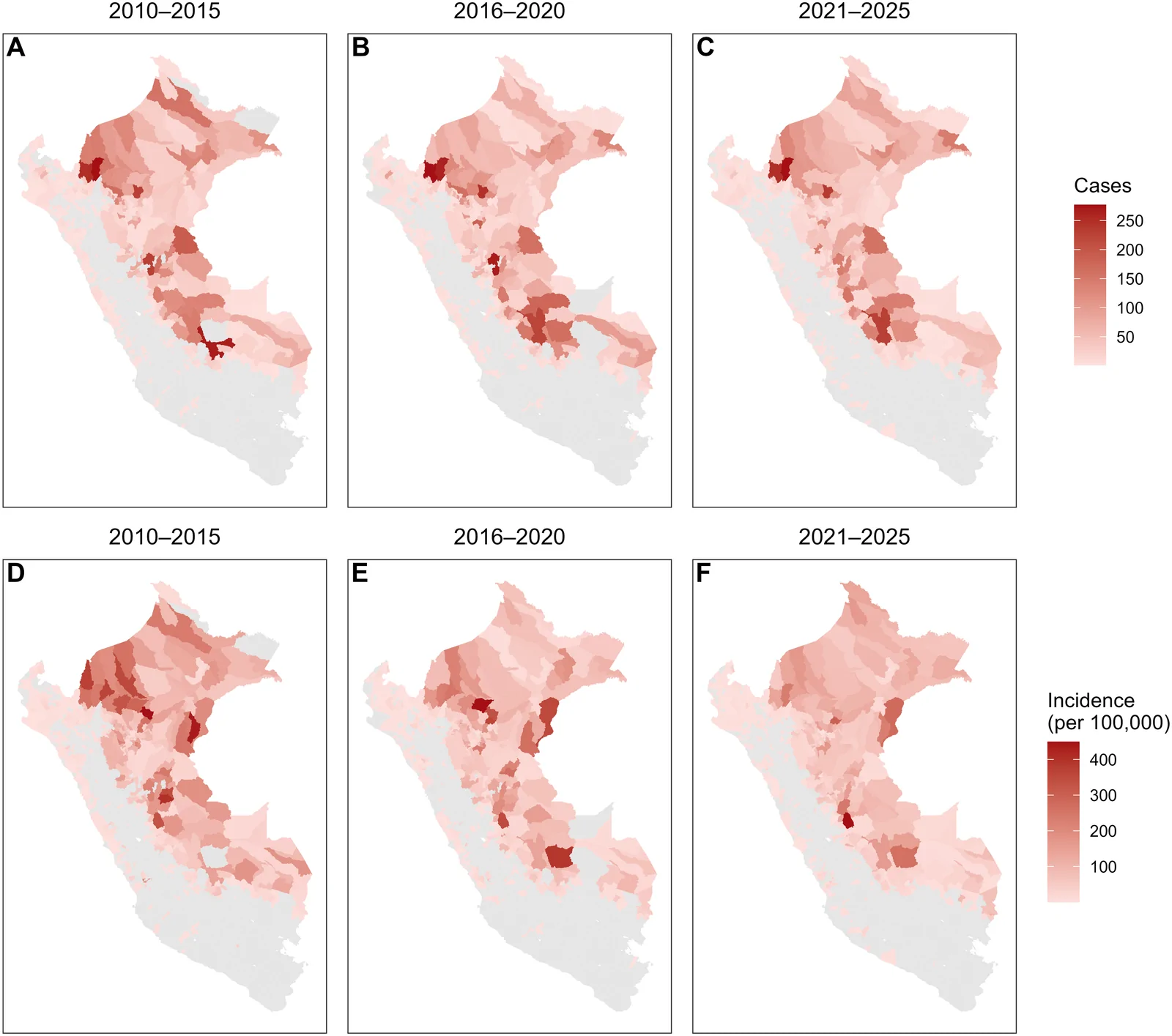
Spatial distribution of SBE cases and incidence in Peru by period (2010–2025). **(A–C)** Total number of reported SBE cases aggregated by department for three periods: 2010–2015, 2016–2020, and 2021–2025. **(D–F)** SBE incidence rates per 100,000 inhabitants for the same periods. Color intensity represents increasing numbers of cases (above panels) and higher incidence rates (below panels). Gray areas indicate departments or periods with insufficient population data to estimate incidence. Administrative boundaries were obtained from the Instituto Geográfico Nacional del Perú (IGN), through the Peruvian National Open Data Platform, using the Límites Departamentales shapefile [39]. The data are available under the Open Data Commons Attribution License (ODC-By).

Across the three study subperiods, the mean annual number of reported cases showed a slight decline, decreasing from 2,192 cases/year during 2010–2015 to 1,994 during 2016–2020 and 1,968 during 2021–2025 (Table 1). Despite this reduction, the spatial distribution of SBE burden remained relatively stable over time, with persistent concentration in Amazonian departments (Fig 3A–C).

Incidence analyses reinforced these spatial disparities (Fig 3D–F; Table 1). During 2010–2015, the highest incidence rates nationwide were observed in Loreto (63.7 cases per 100,000 inhabitants), Ucayali (56.8), San Martín (49.4), Amazonas (46.7), and Madre de Dios (42.7), highlighting the disproportionately high risk of SBE in the Peruvian Amazon. Between 2016 and 2020, incidence declined in several high-burden departments, particularly in Loreto (51.7 cases per 100,000 inhabitants), Ucayali (41.1), Amazonas (38.2), and Madre de Dios (32.7), while remaining relatively stable in San Martín (46.4). During 2021–2025, incidence continued to decrease in Ucayali (37.0) and Madre de Dios (24.2), whereas Loreto (53.5), Amazonas (39.6), and Pasco (25.4) exhibited stable or slightly increasing incidence relative to the previous period. Overall, the highest incidence rates remained concentrated in Amazonian departments throughout the study period, despite moderate temporal fluctuations.

In contrast, coastal and southern departments consistently presented lower incidence rates throughout the study period, generally below 5 cases per 100,000 inhabitants. Extremely low incidence levels were observed in Lima, La Libertad, Lambayeque, Ica, Arequipa, and Apurímac, where incidence frequently remained below 1 case per 100,000 inhabitants across all study periods.

### Spatial clustering and persistence of SBE hotspots

Global Moran’s I revealed statistically significant positive spatial autocorrelation in district-level SBE incidence throughout the study period. Moran’s I values were 0.607 for 2010–2015, 0.525 for 2016–2020, and 0.578 for 2021–2025. Sensitivity analyses using alternative neighborhood definitions (k = 6, k = 8, and Queen contiguity) yielded highly consistent results, with Moran’s I values ranging from 0.503 to 0.614 across all weighting schemes and study periods (S3 Table). Although the number of significant clusters varied according to the spatial weights matrix, the overall hotspot configuration remained remarkably stable, supporting the robustness of the identified spatial patterns.

Local Indicators of Spatial Association (LISA) identified 134, 142, and 167 High–High (HH) districts during 2010–2015, 2016–2020, and 2021–2025, respectively (Fig 4). Under the primary kNN = 4 specification, no statistically significant Low–Low (LL) clusters were detected, whereas 24, 19, and 17 districts were classified as Low–High (LH) outliers and 13, 10, and 12 as High–Low (HL) outliers during the three periods, respectively (S3 Table). A complete period-specific breakdown of Global Moran’s I and LISA classifications under the primary and alternative spatial weights matrices, including HH, LL, LH, HL, and non-significant districts, is presented in S3 Table.

**Fig 4:**
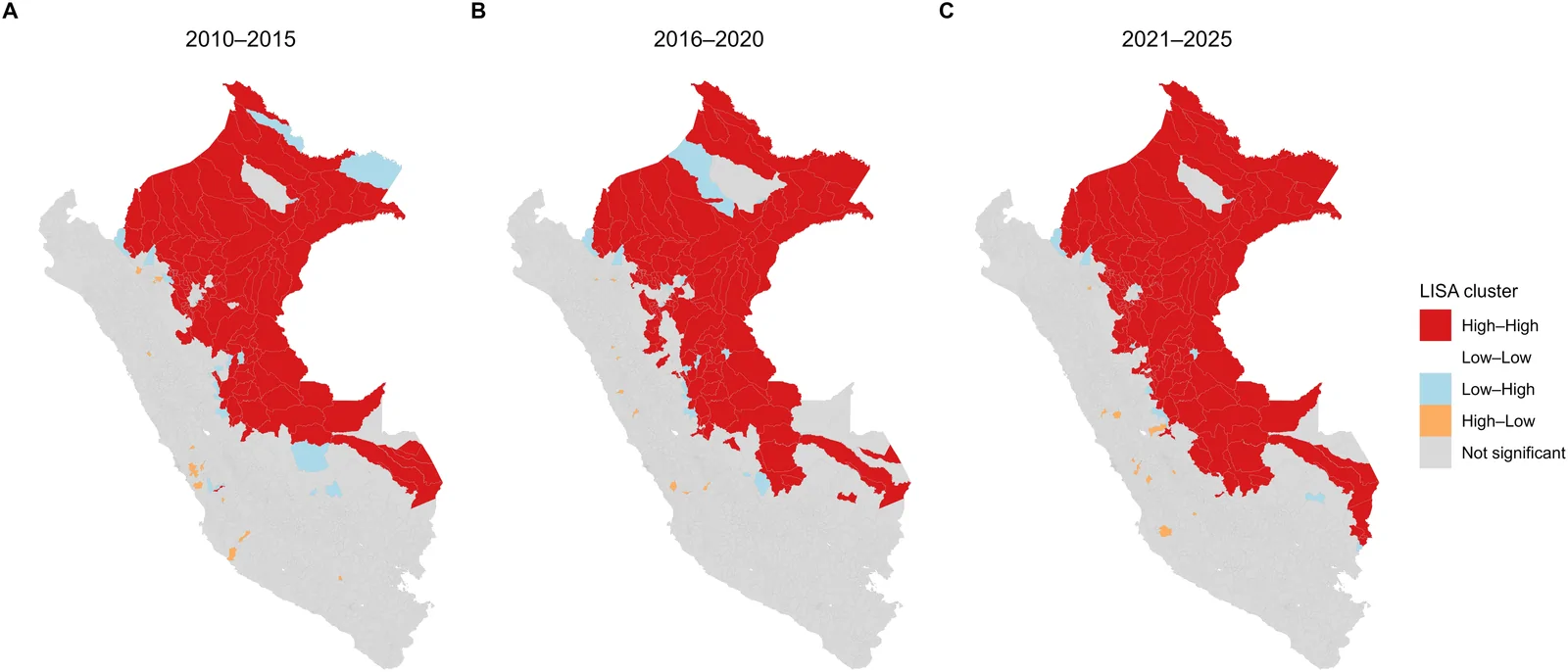
Spatial distribution of Local Indicators of Spatial Association (LISA) clusters of SBE incidence at the district level, Peru, 2010–2025. Panels show significant local spatial clusters for three study periods: **(A)** 2010–2015, **(B)** 2016–2020, and **(C)** 2021–2025. High–High districts (red) represent areas with high incidence surrounded by neighboring districts with similarly high incidence. Low–Low districts (white) indicate areas with low incidence surrounded by low-incidence neighbors. Low–High districts (light blue) correspond to low-incidence districts surrounded by high-incidence neighbors, while High–Low districts (orange) represent high-incidence districts surrounded by low-incidence neighbors. Grey areas indicate districts where local spatial autocorrelation was not statistically significant. Statistical significance was assessed using Local Moran’s I (*p* < 0.05). Under the primary kNN4 spatial weighting scheme, High–High clusters predominated, whereas no statistically significant Low–Low (LL) clusters were identified. Administrative boundaries were obtained from the Instituto Geográfico Nacional del Perú (IGN), through the Peruvian National Open Data Platform, using the Límites Distritales shapefile [39]. The data are available under the Open Data Commons Attribution License (ODC-By).

HH clusters consistently formed a large and spatially contiguous hotspot corridor extending across the northern and central Peruvian Amazon, particularly in Loreto, Amazonas, San Martín, Ucayali, Huánuco, Pasco, and Madre de Dios (Fig 4). The spatial continuity of these hotspots remained across the three study periods despite temporal variation in incidence levels.

A total of 147 districts were classified as persistent hotspots, including 114 districts that formed part of significant HH clusters in all three study periods and 33 districts classified as HH in two periods. In contrast, only 35 districts were identified as transient hotspots, appearing as HH clusters in a single period (S4 Table). Persistent hotspots were concentrated almost exclusively within the Amazon basin, whereas coastal and southern districts were predominantly classified as non-hotspots (Fig 5A).

**Fig 5:**
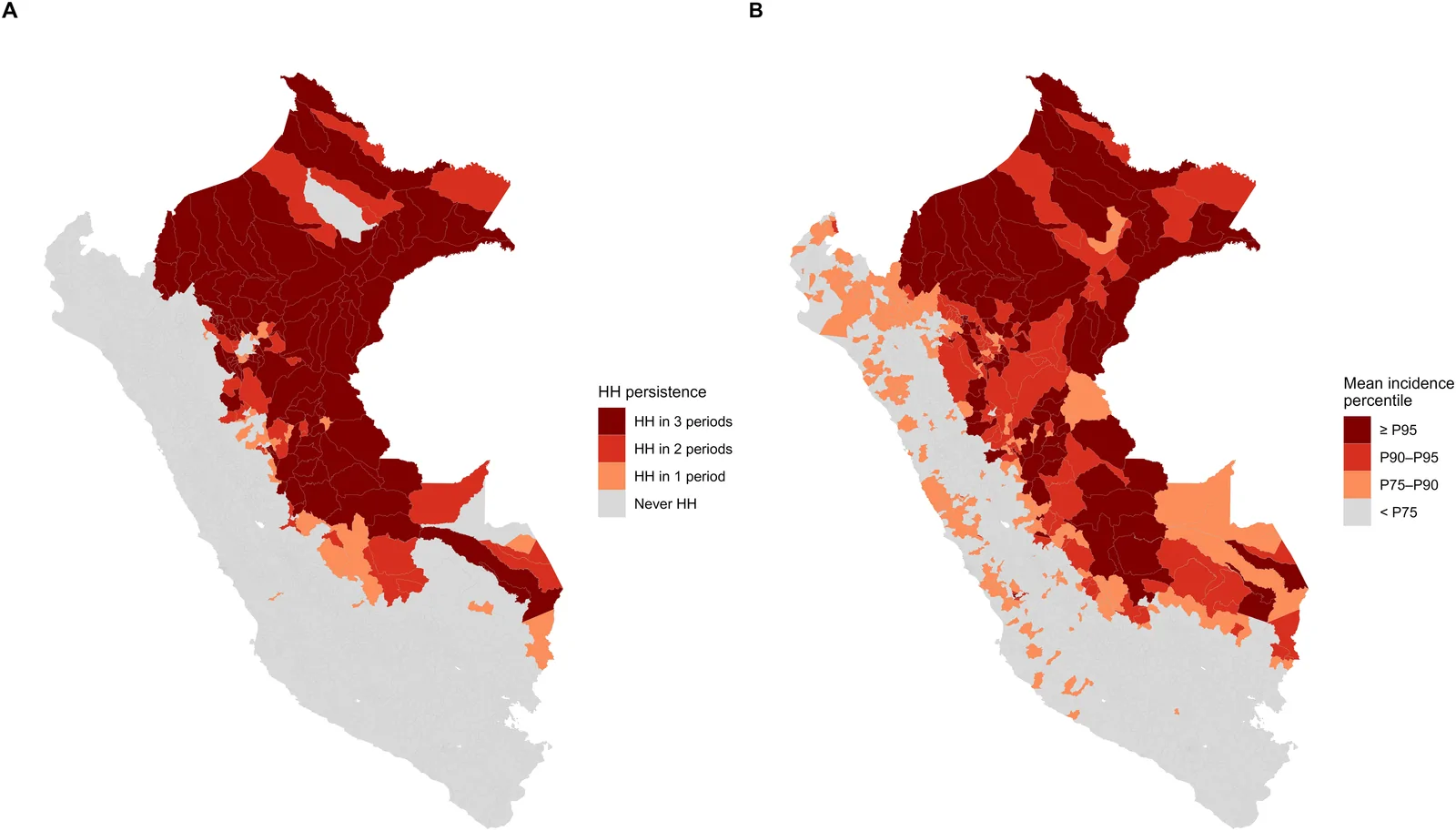
Spatial persistence and long-term incidence burden of snakebite envenoming hotspots in Peru, 2010–2025. **(A)** Persistence of district-level High–High (HH) clusters identified using Local Moran’s I statistics across three study periods (2010–2015, 2016–2020, and 2021–2025). Districts were classified according to the number of periods in which they formed part of a significant HH cluster (HH in 1, 2, or 3 periods). Districts classified as “Never HH” did not belong to a significant HH cluster in any study period. Statistical significance was assessed at *p* < 0.05. **(B)** Long-term district-level SBE incidence, calculated as the mean of the annualized incidence estimates across the three study periods. Percentile thresholds were calculated across all 1,891 districts, including zero-incidence districts, and corresponded to P75 = 1.80, P90 = 24.83, and P95 = 65.56 cases per 100,000 inhabitants. Administrative boundaries were obtained from the Instituto Geográfico Nacional del Perú (IGN), through the Peruvian National Open Data Platform, using the Límites Distritales shapefile [39]. The data are available under the Open Data Commons Attribution License (ODC-By).

To complement the spatial persistence analysis, districts were classified according to long-term incidence burden using the mean annualized incidence across the three study periods (2010–2025) (Fig 5B). Districts within the highest incidence category (≥P95) were predominantly concentrated in persistent hotspot areas: 76 districts (80.0%) belonged to HH clusters in all three periods and an additional 9 districts (9.5%) belonged to HH clusters in two periods. Similarly, among districts within the P90–P95 category, 44 districts (46.8%) were persistent hotspots. In contrast, most districts classified below the P75 threshold (n = 1,418) were never identified as HH clusters (S4 Table).

### Ecological suitability and potential richness of medically important venomous snakes in Peru

Environmental suitability models revealed pronounced spatial heterogeneity in the distribution of medically important venomous snakes across Peru (Fig 6). Suitability maps for the remaining medically important venomous snake species are presented in S3 Fig. *Bothrops atrox*, the species most strongly associated with SBE envenoming in the Peruvian Amazon, exhibited high environmental suitability predominantly in lowland Amazonian districts, particularly within the northern and central Amazon regions (Fig 6A). Suitability values progressively declined toward Andean and coastal regions.

**Fig 6:**
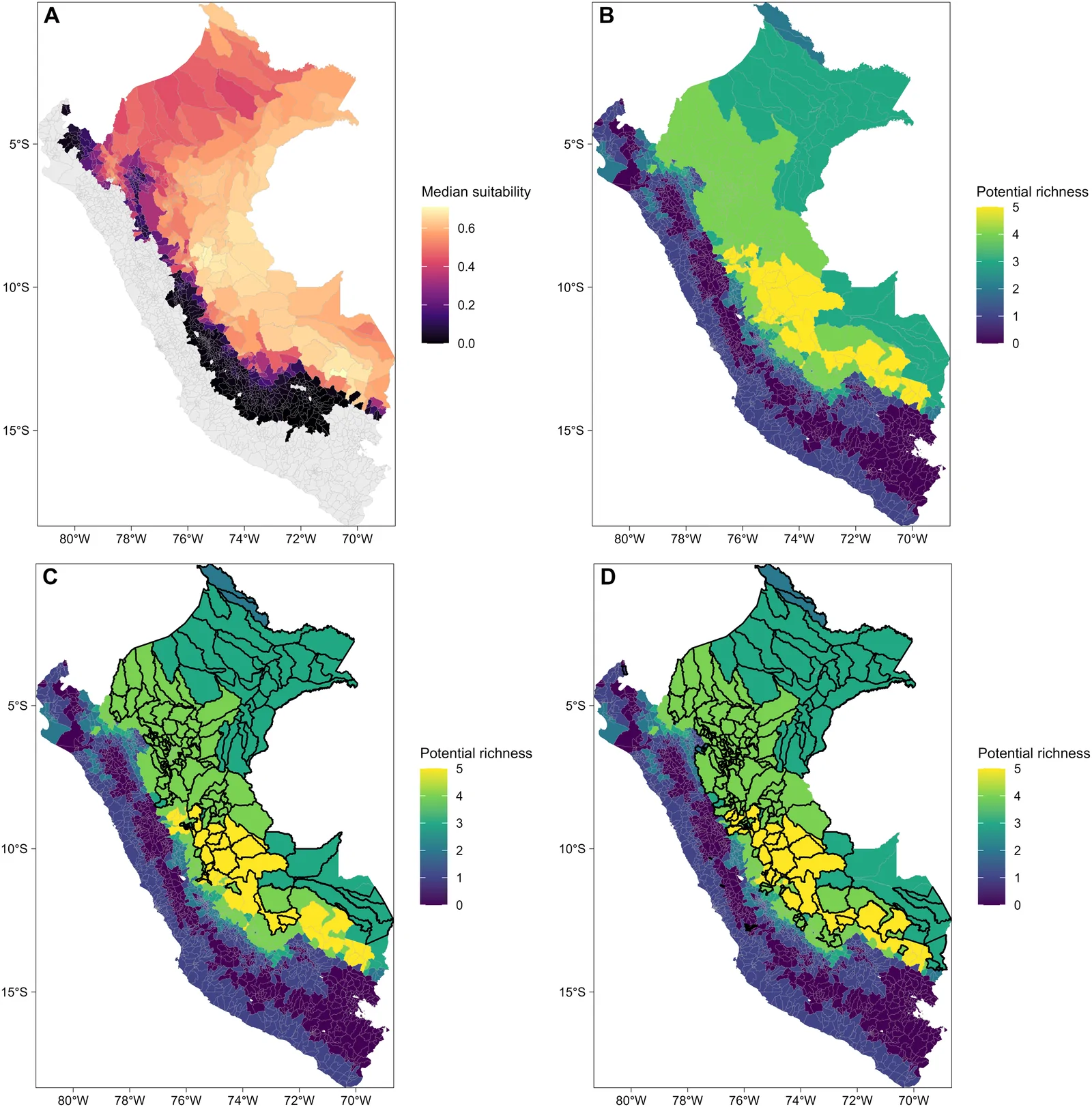
Environmental suitability, potential venomous snake richness, and spatial overlap with persistent SBE hotspots in Peru. **(A)** Environmental suitability of *Bothrops atrox*, represented by median suitability values derived from species distribution models. Higher values indicate environmentally favorable conditions for the occurrence of this species. **(B)** Potential richness of medically important venomous snake species across Peruvian districts, estimated from the overlap of binary distribution models for *Bothrops atrox*, *Bothrops bilineatus*, *Bothrops barnetti*, *Bothrops pictus*, *Bothrops oligolepis*, *Bothrocophias microphthalmus*, and *Lachesis muta*. **(C)** Spatial overlap between potential venomous snake richness and persistent High–High (HH) LISA snakebite envenoming hotspots. Black outlines indicate districts classified as HH clusters in at least two of the three study periods (2010–2015, 2016–2020, and 2021–2025). **(D)** Spatial overlap between potential venomous snake richness and districts with high long-term snakebite incidence. Black outlines indicate districts whose mean snakebite incidence during 2010–2025 was within the upper 10% of the national distribution (P90–P95 and ≥P95). Species distribution model outputs were obtained from the VenomMaps web application [47] and are available under the Creative Commons Attribution 4.0 International License (CC BY 4.0). Administrative boundaries were obtained from the Instituto Geográfico Nacional del Perú (IGN), through the Peruvian National Open Data Platform, using the Límites Distritales shapefile [39]. The administrative boundary data are available under the Open Data Commons Attribution License (ODC-By).

Potential species richness also showed a marked spatial gradient across the country (Fig 6B). Districts with the highest richness values were concentrated mainly in the Amazon basin and eastern Andean foothills, indicating greater potential overlap among medically important venomous snake species in these regions. In contrast, coastal and southern Andean districts generally exhibited low richness values, reflecting more restricted ecological suitability for venomous pitvipers.

The spatial distribution of snakebite envenoming was strongly associated with these ecological patterns. Persistent High–High (HH) LISA hotspots largely coincided with districts characterized by elevated venomous snake richness, particularly in northern and central Amazonia (Fig 6C). Similarly, districts with the highest long-term snakebite incidence (mean incidence ≥ P90 during 2010–2025) were concentrated in areas of high potential richness and environmental suitability (Fig 6D).

Statistical analyses confirmed these spatial associations. Districts classified as persistent HH hotspots exhibited significantly greater potential richness of medically important venomous snake species than non-persistent or non-hotspot districts (Wilcoxon rank-sum test, *W* = 14,235.5, *p* < 0.001). Persistent HH hotspots presented a mean richness of 3.81 species (median = 4), compared with 1.03 species (median = 1) in the remaining districts.

A similar pattern was observed when districts were classified according to long-term snakebite incidence percentiles. Districts within the highest incidence category (P95) showed significantly greater richness than all other districts combined (*W* = 12,304.5, *p* < 0.001), with a mean richness of 3.79 species (median = 4). Likewise, districts classified within the upper P90 incidence class exhibited a mean richness of 3.72 species (median = 4), significantly higher than districts below this threshold (*W* = 20,969.5, *p* < 0.001). When considering the broader P75 category, high-incidence districts still displayed significantly greater richness (mean = 2.65 species; median = 3) than lower-incidence districts (mean = 0.77 species; median = 1; *W* = 120,066.5, *p* < 0.001).

### Socioeconomic inequalities and SBE incidence across Peruvian macroregions

District-level analyses integrating the Human Development Index and SBE incidence during 2017–2024 revealed marked geographic heterogeneity in the relationship between socioeconomic development and SBE burden across Peru (Fig 7). The strongest association was observed in the Northern Amazon, where lower HDI was significantly associated with higher SBE incidence (Spearman’s ρ = -0.42; 95% bootstrap CI: -0.53 to -0.29; *p* < 0.001; *n* = 177). A similar negative association was observed in the Central Amazon, although it was not statistically significant and had a wide confidence interval (ρ = -0.38; 95% CI: -0.75 to 0.12; *p* = 0.138; *n* = 17).

**Fig 7:**
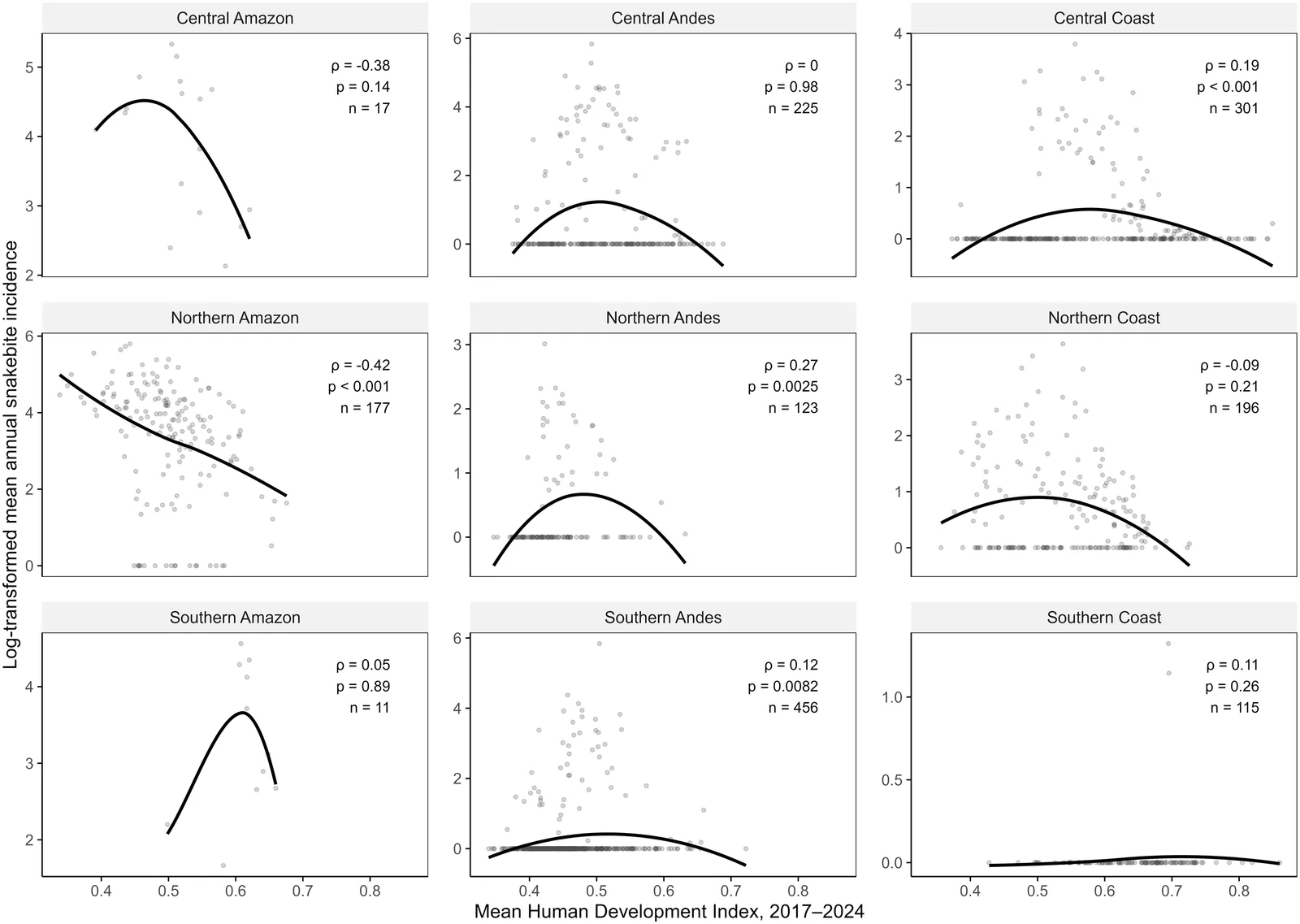
Relationship between district-level human development and SBE incidence across Peruvian macroregions during 2017–2024. Panels show associations between the mean Human Development Index and log-transformed mean annual SBE incidence at the district level. Solid black lines represent LOESS-smoothed trends. Spearman’s correlation coefficient (ρ), *p*-values, and number of districts analyzed (*n*) are shown in each panel.

Weak but statistically significant positive associations were observed in the Central Coast (ρ = 0.19; 95% CI: 0.10 to 0.28; *p* < 0.001; *n* = 301), Northern Andes (ρ = 0.27; 95% CI: 0.12 to 0.40; *p* = 0.002; *n* = 123), and Southern Andes (ρ = 0.12; 95% CI: 0.04 to 0.21; *p* = 0.008; *n* = 456). No statistically significant associations were observed in the Central Andes, Northern Coast, Southern Amazon, or Southern Coast. Complete macroregion-specific estimates, including Spearman’s ρ, bootstrap 95% confidence intervals, p-values, and sample sizes, are presented in S5 Table and S4 Fig. The Southern Coast estimate should be interpreted cautiously because 113 of the 115 eligible districts had zero SBE incidence, resulting in insufficient variation to estimate a stable bootstrap confidence interval.

## Discussion

The present study provides the first nationwide multiscale spatiotemporal assessment of SBE in Peru between 2010 and 2025, revealing both a strong concentration of reported SBE cases (burden) and persistently high incidence rates in Amazonian regions. Both descriptive analyses and spatial autocorrelation approaches consistently demonstrated that SBE incidence in Peru is not randomly distributed, but instead organized into stable geographic hotspots predominantly located in northern and central Amazonian districts. These findings reinforce growing evidence that SBE should be understood as a territorially structured neglected tropical disease associated with interacting ecological and socioeconomic conditions [2,15,51]. Recent studies further suggest that SBE patterns emerge from coupled socioecological systems in which climate variability, land-use change, forest structure, and human occupation interact to influence exposure risk across tropical landscapes [56–58].

The marked concentration of reported SBE cases in departments such as Loreto, San Martín, and Ucayali reflects epidemiological patterns widely reported across tropical forest regions, where SBE disproportionately affects rural populations engaged in agriculture, forestry, fishing, extractive activities, and other forest-dependent livelihoods [9,51,59]. Together, these departments accounted for more than 60% of all reported SBE cases during the study period, representing the largest SBE burden in Peru and highlighting the central role of the Amazon basin in the national epidemiology of SBE. These departments also exhibited some of the highest incidence rates nationwide, indicating both a high disease burden and elevated population-level risk. Similar hotspot configurations have been documented in Brazil, Colombia, Mexico, and Nepal, where SBE incidence is strongly associated with environmental suitability, rurality, and socioeconomic vulnerability [25,60,61].

The high SBE incidence observed in Amazonian Peru likely results from the interaction of ecological, environmental, and socioeconomic factors. Tropical forest ecosystems provide highly suitable habitats for medically important venomous snakes, particularly species of the genus *Bothrops*, which are responsible for most SBE cases in the Peruvian Amazon. Simultaneously, agricultural expansion, forest degradation, extractive activities, road opening, and increasing human occupation of forested landscapes may intensify human–snake encounters and consequently increase exposure risk [4,14].

Climate and hydrological conditions also likely play an important role in shaping SBE dynamics in Amazonian Peru. Previous studies have demonstrated that rainfall variability, flooding events, and environmental change can alter snake behavior, habitat suitability, prey availability, agricultural practices, and human mobility, thereby influencing exposure risk [25,62]. In the present study, SBE incidence was consistently higher during wetter months, supporting the influence of seasonal climatic conditions on SBE occurrence. Comparable seasonal relationships have been documented in both the Brazilian Amazon and Bangladesh, where increased precipitation was associated with higher snake activity and increased incidence among rural populations, particularly adult males involved in outdoor occupations [56,63]. Together, these findings reinforce the importance of hydroclimatic seasonality as an important driver of SBE dynamics across tropical regions.

Although the national number of reported cases showed a gradual decline between 2010 and 2025, the spatial structure of SBE incidence remained remarkably stable across study periods. This finding suggests that the territorial determinants underlying SBE transmission have changed relatively little over the last 15 years. In particular, the persistence of high-incidence districts along the Amazonian corridor indicates that SBE in Peru behaves as a structurally embedded territorial health problem rather than as an episodic or randomly distributed event. The continuity of hotspot areas across multiple periods reinforces the existence of long-term ecological and socioeconomic conditions that sustain elevated SBE risk in Amazonian territories.

The marked reduction observed during 2020 and 2021 should, however, be interpreted cautiously. Although this decline may partially reflect a true reduction in SBE exposure during the COVID-19 pandemic, it may also be associated with reduced healthcare-seeking behavior, mobility restrictions, disruptions in healthcare access, and temporary limitations in epidemiological surveillance and notification systems during the pandemic period [64].

The significant positive spatial autocorrelation identified by Global Moran’s I further supports the existence of geographically structured SBE risk in Peru. Likewise, the LISA analysis demonstrated that High–High clusters were strongly concentrated in Amazonian districts. Under the primary kNN4 spatial weighting scheme, no statistically significant Low–Low (LL) clusters were identified. Most coastal and southern districts were classified as non-significant areas or isolated spatial outliers, despite consistently exhibiting the lowest incidence rates observed in Peru. This marked spatial configuration highlights the strong epidemiological contrast between Amazonian and non-Amazonian territories. Coastal departments such as Lima, Ica, and La Libertad consistently exhibited very low incidence rates throughout the study period, reflecting major ecological, demographic, and socioeconomic differences relative to tropical forest regions.

One of the most important findings of this study was the identification of persistent hotspot corridors in northern and central Amazonian Peru. Persistent hotspots have important implications for public health surveillance and antivenom allocation strategies because they indicate territories where elevated transmission risk remains stable over time. Rather than relying exclusively on national-level indicators, spatially explicit approaches may help prioritize districts with recurrent high transmission and optimize the distribution of healthcare resources. Persistent hotspot districts may require strengthened epidemiological surveillance systems, improved antivenom availability, faster transportation routes to healthcare facilities, and prevention campaigns directed toward high-risk occupational groups.

Recent spatial modeling studies have emphasized that combining epidemiological surveillance with ecological suitability models may substantially improve territorial prioritization for antivenom distribution, prevention campaigns, and healthcare preparedness, particularly under scenarios of environmental and climatic change [58,65]. These findings reinforce the importance of incorporating spatial epidemiology into SBE prevention programs, especially in geographically isolated Amazonian settings where logistical constraints frequently delay access to life-saving treatment [66].

Moreover, the strong spatial overlap observed between persistent epidemiological hotspots, high environmental suitability for *Bothrops atrox*, and elevated potential venomous snake richness suggests that ecological and socioeconomic factors may be associated with the spatial distribution of SBE burden in Peru. This pattern was particularly evident in northern Amazonian districts, where lower levels of human development were associated with higher SBE incidence [2,51]. Together, these findings suggest that persistent SBE hotspots tend to occur in territories characterized by both high environmental suitability and less favorable socioeconomic conditions. However, the present analyses do not allow causal inference regarding the relative contribution of these factors to hotspot persistence.

Importantly, SBE burden in the Amazon cannot be interpreted solely as a consequence of environmental exposure. Structural inequalities and limited healthcare accessibility likely contribute substantially to both exposure risk and delayed treatment. The negative association identified between HDI and SBE incidence in northern Amazonian districts reinforces the recognition of SBE as a disease closely linked to social vulnerability and territorial inequalities. Previous studies have emphasized that SBE disproportionately affects historically neglected rural populations and should therefore be understood simultaneously as a tropical disease, a poverty-related condition, and a territorial health inequity problem [5,59]. In remote Amazonian areas, where river transport often represents the only means of accessing healthcare services, delays in antivenom administration remain a major challenge and may contribute to underreporting, severe complications, disability, and mortality. Moreover, because healthcare accessibility and epidemiological surveillance capacity are highly heterogeneous across Peru, the observed concentration of SBE in Amazonian territories may still represent a conservative estimate of the true burden in these regions.

In contrast, the weak or slightly positive correlations observed in some Andean and Coastal macroregions should be interpreted cautiously. In regions with overall low SBE burden, small increases in incidence may become proportionally more detectable in districts with better healthcare infrastructure and surveillance systems, potentially generating weak positive correlations between HDI and reported incidence. These findings suggest that the relationship between socioeconomic development and SBE incidence is spatially heterogeneous across Peru and influenced not only by exposure risk, but also by healthcare accessibility and notification efficiency.

Overall, the results demonstrate that SBE in Peru is simultaneously shaped by interacting ecological and socioeconomic determinants. Amazonian territories concentrated both the largest number of reported SBE cases and the highest incidence rates throughout the study period. The persistence of geographically stable hotspots throughout the 2010–2025 study period underscores the urgent need for spatially targeted and territorially adapted public health interventions in the Peruvian Amazon. Although national incidence showed a gradual decline over time, the long-term maintenance of High–High clusters in Amazonian districts indicates that SBE remains a structurally persistent territorial health problem.

Future studies integrating environmental variables, land-use dynamics, climate variability, healthcare accessibility, and socioeconomic indicators may substantially improve the understanding of SBE risk determinants and support predictive risk mapping under ongoing scenarios of environmental transformation and climate change. In this context, combining spatial epidemiology, ecological suitability modeling, and socioeconomic analyses may contribute to the development of early-warning systems and more efficient territorial allocation of prevention, surveillance, and antivenom distribution strategies in high-risk Amazonian regions [2,67].

### Limitations

This study provides the first nationwide multiscale spatiotemporal assessment of SBE incidence in Peru between 2010 and 2025. Nevertheless, some limitations should be considered when interpreting the results. The surveillance database consisted exclusively of aggregated annual records and did not include information on snake species, clinical severity, occupation, exact geographic coordinates, or precise dates of occurrence. Consequently, the analyses were conducted using administrative spatial units rather than individual-level or point-based data. In addition, the study likely underestimates the true burden of SBE, particularly in remote Amazonian, Indigenous, and riverine communities where healthcare accessibility is limited and traditional medicine frequently represents the first therapeutic response before formal medical care is sought.

Potential underreporting is also unlikely to be spatially homogeneous across Peru. Districts with greater healthcare infrastructure and surveillance capacity may present more complete notification systems than geographically isolated Amazonian territories, potentially introducing spatial surveillance bias. An additional limitation concerns the interpretation of temporal trends during the COVID-19 pandemic period (2020–2021). Although the observed decline in reported SBE incidence may partially reflect changes in exposure patterns, it could also have been influenced by reduced healthcare-seeking behavior, mobility restrictions, disruptions in healthcare access, and temporary limitations in epidemiological surveillance and notification systems. Consequently, incidence estimates during this period should be interpreted with caution, as they may not fully reflect underlying SBE occurrence.

Furthermore, the use of district-level aggregated data may introduce spatial aggregation effects associated with the Modifiable Areal Unit Problem (MAUP), potentially masking local heterogeneities and influencing the spatial configuration of identified clusters. Likewise, associations observed between Human Development Index (HDI), ecological suitability, and SBE incidence should not be interpreted as direct causal relationships at the individual level, because the analyses are subject to potential ecological fallacy.

Species distribution models estimate environmental suitability rather than snake abundance or envenoming risk and may be influenced by spatial biases in occurrence records and modeling choices. Moreover, relevant variables, including occupational exposure, land-use dynamics, healthcare access, referral capacity, and antivenom availability, could not be consistently incorporated at the national scale. Despite these limitations, the nationwide scope, long temporal series, and integration of spatial epidemiology, ecological suitability, and socioeconomic analyses represent an important contribution to understanding the territorial dynamics of SBE in Peru.

## Conclusions

This nationwide spatiotemporal analysis demonstrated that SBE in Peru is strongly concentrated in the Amazon region and organized into geographically persistent high-risk territories, particularly in northern and central Amazonian districts. Amazonian territories concentrated both the largest number of reported SBE cases and the highest incidence rates throughout the study period. Although national incidence showed a slight decline between 2010 and 2025, the spatial structure of SBE incidence remained remarkably stable over time, indicating that high-risk areas are structurally persistent rather than randomly distributed.

The strong spatial overlap between recurrent hotspot districts, environmental suitability for medically important venomous snakes, and socioeconomically vulnerable territories indicates that SBE incidence is spatially associated with ecological suitability and socioeconomic vulnerability. These associations should not be interpreted as evidence of direct causal relationships. These findings reinforce the recognition of SBE as a territorially structured neglected tropical disease strongly associated with social vulnerability and environmental exposure in the Amazon basin.

By integrating long-term spatial epidemiology, hotspot persistence analyses, ecological suitability modeling, and socioeconomic indicators at a national scale, this study provides an important baseline for understanding the territorial dynamics of SBE in Peru. Persistent hotspot corridors identified in the Peruvian Amazon should be considered priority territories for strengthened epidemiological surveillance, decentralized antivenom allocation, improved healthcare preparedness and access, and geographically targeted prevention strategies focused on vulnerable rural and forest-dependent populations.

## Supporting information

S1 Table Classification of Peruvian departments into macroregions used in temporal and socioeconomic analyses. Classification scheme used to group Peruvian departments into Amazonian, Andean, and Coastal macroregions for temporal trend analyses and socioeconomic assessments.
(DOCX)

S2 Table Temporal trend statistics of snakebite incidence across national and macroregional scales in Peru, 2010–2025. Annual Percent Change (APC), linear regression statistics, and Mann–Kendall trend analyses for national and macroregional snakebite incidence rates.
(DOCX)

S3 Table Sensitivity analysis of Global Moran’s I and LISA clusters under alternative spatial weighting schemes. Sensitivity analyses using alternative neighborhood definitions (*k* = 4, 6, and 8; Queen contiguity) yielded highly consistent results. Global Moran’s I remained strongly positive and highly significant across all weighting schemes and study periods (*I* = 0.503–0.614; pseudo-*p* = 0.001).
(DOCX)

S4 Table Distribution of districts according to hotspot persistence and incidence percentile classes, Peru, 2010–2025. HH denotes districts classified as statistically significant High–High clusters in Local Indicators of Spatial Association (LISA) analyses.
(DOCX)

S5 Table Macroregion-specific associations between district-level Human Development Index and snakebite envenoming incidence in Peru, 2017–2024. Spearman’s rank correlation coefficients, 95% bootstrap confidence intervals, p-values, and numbers of districts analyzed are reported for each Peruvian macroregion.
(DOCX)

S1 Fig Annual snakebite incidence across Peruvian macroregions, 2010–2025. Annual snakebite incidence per 100,000 inhabitants across the major Peruvian macroregions between 2010 and 2025. Panels represent the Amazon, Andes, and Coast regions, subdivided into northern, central, and southern sectors.
(TIFF)

S2 Fig Seasonal monthly snakebite incidence across Peruvian macroregions. Mean monthly snakebite incidence per 100,000 inhabitants across the major Peruvian macroregions based on the 2010–2025 period. Seasonal peaks were generally observed during the first months of the year, particularly in Amazonian regions.
(TIFF)

S3 Fig Environmental suitability of medically important venomous snake species in Peru derived from species distribution models. Environmental suitability maps for medically important venomous snake species occurring in Peru, represented by median suitability values derived from species distribution models. Panels correspond to: (A) *Bothrops bilineatus*, (B) *Bothrops barnetti*, (C) *Bothrops pictus*, (D) *Bothrops oligolepis*, (E) *Bothrocophias microphthalmus*, and (F) *Lachesis muta*. Species distribution model outputs were obtained from the VenomMaps web application [47] and are available under the Creative Commons Attribution 4.0 International License (CC BY 4.0). Administrative boundaries were obtained from the Instituto Geográfico Nacional del Perú (IGN), through the Peruvian National Open Data Platform, using the Límites Distritales shapefile [39] (direct dataset link: https://www.datosabiertos.gob.pe/dataset/limites-departamentales/resource/a43e17c8-fa37-463d-aa7e-2ce2a272491b). The administrative boundary data are available under the Open Data Commons Attribution License (ODC-By) (license: https://opendatacommons.org/licenses/by/1-0/). Attribution: Instituto Geográfico Nacional del Perú (IGN), 2022.
(TIFF)

S4 FigRelationship between Human Development Index and snakebite incidence across Peruvian macroregions.Macroregion-specific associations between district-level Human Development Index (HDI) and mean annual snakebite incidence during 2017–2024. Districts with populations below 1,000 inhabitants were excluded to reduce instability associated with very small population sizes. Points represent Spearman’s rank correlation coefficients (ρ), and horizontal lines represent 95% bootstrap confidence intervals. The vertical dashed line indicates ρ=0. Values of ρ, *p*-values, and the number of districts analyzed (*n*) are shown for each macroregion.(TIFF)
