# Supplementary material for: Persistent spatial hotspots and socioeconomic inequalities of snakebite envenoming in Peru: A nationwide spatiotemporal analysis, 2010–2025
Source: PLoS Negl Trop Dis. 2026 Sep 24;20(9):e0014747. doi: 10.1371/journal.pntd.0014747 (PMC13623120; doi:10.1371/journal.pntd.0014747)
Supplement: Supplementary file 1 [file pntd.0014747.s001.docx]

**S1 Table. Classification of Peruvian departments into macroregions used in temporal and socioeconomic analyses.**

| Zone | Sector | Macroregion | Departments |
| --- | --- | --- | --- |
| Amazon | Northern | Northern Amazon | Amazonas, Loreto, San Martín |
| Amazon | Central | Central Amazon | Ucayali |
| Amazon | Southern | Southern Amazon | Madre de Dios |
| Andes | Northern | Northern Andes | Cajamarca |
| Andes | Central | Central Andes | Huánuco, Pasco, Junín |
| Andes | Southern | Southern Andes | Huancavelica, Ayacucho, Apurímac, Cusco, Puno |
| Coast | Northern | Northern Coast | Tumbes, Piura, Lambayeque, La Libertad |
| Coast | Central | Central Coast | Ancash, Lima, Callao*, Ica |
| Coast | Southern | Southern Coast | Arequipa, Moquegua, Tacna |

*Callao corresponds to the Constitutional Province of Callao and is not classified as a

department.
