# Supplementary material for: Persistent spatial hotspots and socioeconomic inequalities of snakebite envenoming in Peru: A nationwide spatiotemporal analysis, 2010–2025
Source: PLoS Negl Trop Dis. 2026 Sep 24;20(9):e0014747. doi: 10.1371/journal.pntd.0014747 (PMC13623120; doi:10.1371/journal.pntd.0014747)
Supplement: Supplementary file 2 [file pntd.0014747.s002.docx]

### **S2 Table. Temporal trend statistics of snakebite incidence across national and macroregional scales in Peru, 2010–2025.**

| **Scale** | **Region** | **APC (%)** | **Linear model p-value** | **R²** | **Mann–Kendall τ** | **Mann–Kendall p-value** |
| --- | --- | --- | --- | --- | --- | --- |
| National | Peru | -1.76 | 0.0018 | 0.512 | -0.65 | <0.001 |
| Macroregion | Northern Amazon | -2.01 | <0.001 | 0.566 | -0.667 | <0.001 |
| Macroregion | Central Amazon | -2.25 | 0.0042 | 0.454 | -0.35 | 0.065 |
| Macroregion | Southern Amazon | -4.19 | 0.0107 | 0.382 | -0.383 | 0.043 |
| Macroregion | Northern Andes | -7.14 | 0.0023 | 0.497 | -0.393 | 0.038 |
| Macroregion | Central Andes | 0.62 | 0.368 | 0.058 | 0.183 | 0.344 |
| Macroregion | Southern Andes | 0.45 | 0.812 | 0.004 | 0.41 | 0.031 |
| Macroregion | Northern Coast | -2.93 | 0.16 | 0.136 | -0.317 | 0.096 |
| Macroregion | Central Coast | -2.94 | 0.122 | 0.162 | -0.25 | 0.192 |
| Macroregion | Southern Coast | 3.06 | 0.614 | 0.019 | 0.085 | 0.715 |

### APC: Annual Percent Change. Mann–Kendall τ values and associated p-values indicate the direction and significance of monotonic temporal trends.
