# Supplementary material for: Persistent spatial hotspots and socioeconomic inequalities of snakebite envenoming in Peru: A nationwide spatiotemporal analysis, 2010–2025
Source: PLoS Negl Trop Dis. 2026 Sep 24;20(9):e0014747. doi: 10.1371/journal.pntd.0014747 (PMC13623120; doi:10.1371/journal.pntd.0014747)
Supplement: Supplementary file 3 [file pntd.0014747.s003.docx]

**S3 Table. Sensitivity analysis of Global Moran’s I and district-level LISA classifications under alternative spatial weights matrices, Peru, 2010–2025.**

| **Period** | **Spatial weights matrix** | **Global Moran’s I** | **Global pseudo-p** | **HH** | **LL** | **LH** | **HL** | **NS** |
| --- | --- | --- | --- | --- | --- | --- | --- | --- |
| 2010–2015 | kNN4ᵃ | 0.607 | 0.001 | 134 | 0 | 24 | 13 | 1,720 |
| 2010–2015 | kNN6 | 0.599 | 0.001 | 153 | 45 | 28 | 10 | 1,655 |
| 2010–2015 | kNN8 | 0.568 | 0.001 | 165 | 397 | 30 | 6 | 1,293 |
| 2010–2015 | Queen | 0.614 | 0.001 | 142 | 20 | 20 | 8 | 1,701 |
| 2016–2020 | kNN4ᵃ | 0.525 | 0.001 | 142 | 0 | 19 | 10 | 1,720 |
| 2016–2020 | kNN6 | 0.512 | 0.001 | 168 | 60 | 28 | 5 | 1,630 |
| 2016–2020 | kNN8 | 0.503 | 0.001 | 172 | 386 | 31 | 4 | 1,298 |
| 2016–2020 | Queen | 0.532 | 0.001 | 151 | 26 | 19 | 6 | 1,689 |
| 2021–2025 | kNN4ᵃ | 0.578 | 0.001 | 167 | 0 | 17 | 12 | 1,695 |
| 2021–2025 | kNN6 | 0.561 | 0.001 | 185 | 46 | 20 | 10 | 1,630 |
| 2021–2025 | kNN8 | 0.538 | 0.001 | 193 | 348 | 24 | 10 | 1,316 |
| 2021–2025 | Queen | 0.613 | 0.001 | 174 | 27 | 13 | 7 | 1,670 |

**Note:** HH, High–High; LL, Low–Low; LH, Low–High; HL, High–Low; NS, not statistically significant. Global Moran’s I significance was assessed using 999 Monte Carlo permutations. LISA classifications were based on permutation-derived local pseudo-p values, with statistical significance defined as pseudo-p < 0.05. Counts refer to the complete set of 1,891 Peruvian districts. Spatial weights were row-standardized. The kNN4 matrix was the primary specification, whereas kNN6, kNN8, and first-order Queen contiguity were used for sensitivity analyses.

ᵃ Primary spatial weights specification.
