# Supplementary material for: Persistent spatial hotspots and socioeconomic inequalities of snakebite envenoming in Peru: A nationwide spatiotemporal analysis, 2010–2025
Source: PLoS Negl Trop Dis. 2026 Sep 24;20(9):e0014747. doi: 10.1371/journal.pntd.0014747 (PMC13623120; doi:10.1371/journal.pntd.0014747)
Supplement: Supplementary file 4 [file pntd.0014747.s004.docx]

**S4 Table. Distribution of districts according to hotspot persistence and incidence percentile classes, Peru, 2010–2025.**

| **Hotspot persistence** | **≥P95** | **P90–<P95** | **P75–<P90** | **<P75** | **Total** |
| --- | --- | --- | --- | --- | --- |
| HH in 3 periods | 76 | 32 | 6 | 0 | 114 |
| HH in 2 periods | 9 | 12 | 12 | 0 | 33 |
| HH in 1 period | 2 | 17 | 16 | 0 | 35 |
| Never HH | 8 | 34 | 249 | 1,418 | 1,709 |
| Total | 95 | 95 | 283 | 1,418 | 1,891 |

**Note:** HH denotes districts classified as statistically significant High–High clusters in period-specific LISA analyses. Long-term incidence was calculated as the mean of the annualized district-level incidence estimates across the three study periods. Percentile thresholds were calculated using all 1,891 districts, including 1,197 districts with zero long-term mean incidence. The resulting thresholds were P75 = 1.80, P90 = 24.83, and P95 = 65.56 cases per 100,000 inhabitants. Categories were defined as <P75, ≥P75 to <P90, ≥P90 to <P95, and ≥P95. Persistent hotspots corresponded to districts classified as HH in two or more study periods.
