# Supplementary material for: Persistent spatial hotspots and socioeconomic inequalities of snakebite envenoming in Peru: A nationwide spatiotemporal analysis, 2010–2025
Source: PLoS Negl Trop Dis. 2026 Sep 24;20(9):e0014747. doi: 10.1371/journal.pntd.0014747 (PMC13623120; doi:10.1371/journal.pntd.0014747)
Supplement: Supplementary file 5 [file pntd.0014747.s005.docx]

**S5 Table. Macroregion-specific associations between district-level Human Development Index and snakebite envenoming incidence in Peru, 2017–2024.**

| **Macroregion** | **n** | **Spearman’s ρ** | **95% bootstrap CI** | **p-value** |
| --- | --- | --- | --- | --- |
| Central Amazon | 17 | −0.38 | −0.75 to 0.12 | 0.138 |
| Central Andes | 225 | 0 | −0.12 to 0.11 | 0.977 |
| Central Coast | 301 | 0.19 | 0.10 to 0.28 | <0.001 |
| Northern Amazon | 177 | −0.42 | −0.53 to −0.29 | <0.001 |
| Northern Andes | 123 | 0.27 | 0.12 to 0.40 | 0.002 |
| Northern Coast | 196 | −0.09 | −0.23 to 0.05 | 0.205 |
| Southern Amazon | 11 | 0.05 | −0.80 to 0.62 | 0.894 |
| Southern Andes | 456 | 0.12 | 0.04 to 0.21 | 0.008 |
| Southern Coast | 115 | 0.11 | Not estimableᵃ | 0.259 |

**Note:** Associations were assessed using two-sided Spearman rank correlations. Districts with a mean population below 1,000 inhabitants or missing HDI or incidence data were excluded. HDI and SBE incidence were summarized across the 2017–2024 period. Confidence intervals were estimated using 5,000 non-parametric bootstrap resamples. Estimates for macroregions with small sample sizes or limited incidence variability should be interpreted cautiously.

ᵃ The bootstrap confidence interval was not reported for the Southern Coast because 113 of 115 districts had zero SBE incidence, resulting in only three unique incidence values and undefined correlation estimates in a substantial proportion of bootstrap resamples.

**Abbreviations:** CI, confidence interval; HDI, Human Development Index; SBE, snakebite envenoming; ρ, Spearman’s rank correlation coefficient.
